# MRI and ^18^F-fluorothymidine PET-MR for the early evaluation of renal and bone marrow effects in ^177^Lu-DOTATATE therapy

**DOI:** 10.1530/EO-25-0036

**Published:** 2026-03-06

**Authors:** Angeliki Dimopoulou Creusen, Edvin Johansson, Per Hagmar, Håkan Ahlström, Dan Granberg

**Affiliations:** ^1^ Radiology, Department of Surgical Sciences, Uppsala University, Uppsala, Sweden; ^2^ Antaros Medical AB, Mölndal, Sweden; ^3^ Department of Molecular Medicine and Surgery, Karolinska Institute, Stockholm, Sweden

**Keywords:** ^18^F-FLT PET-MR, NET, ^177^Lu-DOTATATE treatment, bone-marrow fat conversion, peptide receptor radionuclide therapy

## Abstract

**Background:**

This study aimed to evaluate whether early renal and bone marrow effects – previously identified through laboratory tests – can be detected using MRI and ^18^F-FLT PET-MR in neuroendocrine tumor patients treated with ^177^Lu-DOTATATE. Despite known early changes, precise clinical tools to monitor these effects remain limited.

**Methods:**

Ten patients with metastatic NETs, scheduled to initiate at least four treatment cycles of ^177^Lu-DOTATATE, were included. MRI was performed at all designated treatment time points, whereas F-FLT PET was acquired only at baseline and on day 2 following treatment initiation. Bone marrow (BM) fat conversion, proliferative activity, and renal effects were studied with MRI and ^18^F-FLT PET-MR. Laboratory tests for hematology and kidney and liver function were taken.

**Results:**

Six patients completed the full imaging protocol with MRI, of whom ^18^F-FLT PET-MR examinations were performed in two patients. All subjects demonstrated increased BM fat content during the study interval, with a mean of +12.8%. The magnitude of the increase varied substantially, from +3.8% to +23.5%. There was a strong correlation between increased BM fat content and reduced peripheral blood cell counts. Decreased FLT uptake was found on day 2 after treatment. An increase in renal arterial flow and parenchymal volume was detected during the first week after treatment. No sustained renal effects were observed.

**Conclusions:**

The hematological effects observed with laboratory tests after ^177^Lu-DOTATATE treatment in neuroendocrine tumor patients can be detected by MRI as an increase in bone marrow fat content. This finding was supported by decreased FLT uptake on ^18^F-FLT PET-MR, indicating decreased bone marrow cell proliferation. MRI can also identify renal effects associated with this therapy.

## Background

Two primary considerations for the optimal treatment of cancer patients are their renal and bone marrow (BM) functions. Selection of the proper treatment regimen requires a functional assessment before treatment start and consideration of the potential detrimental effect the treatment may have on renal and BM function (1, 2, 3). High-precision methods that can be used to study renal and BM protection during treatments and are correlated to long-term outcomes still need to be included.

For BM, magnetic resonance imaging (MRI) can sensitively determine fat fraction (4); an increase in fat could be an early indicator of reduced hematopoietic capacity and a useful biomarker. In addition to the MRI methods, a positron emission tomography (PET) tracer can also be used as a marker of proliferation in BM. 3′-deoxy-3′-^18^F-fluorothymidine (^18^F-FLT) is phosphorylated by thymidine-kinase-1 (TK-1) and trapped inside the proliferating cells during the S phase of the cell cycle, providing an indirect measure of proliferative activity (5).

Neuroendocrine tumors (NETs) are often malignant and metastatic at diagnosis, limiting curative surgery. Treatment options include somatostatin analogs, chemotherapy, and targeted agents, such as everolimus and sunitinib. Because most NETs overexpress somatostatin receptors, peptide receptor radionuclide therapy (PRRT) using radiolabeled analogs has shown promising results for over two decades (6, 7, 8, 9, 10). PRRT delivers radionuclides (e.g., indium-111, yttrium-90, and lutetium-177) bound to peptides targeting tumor receptors, but kidneys and bone marrow are dose-limiting organs (1, 2, 11). ^177^Lu-DOTATATE (Lutathera®), approved in 2017–2018 based on the NETTER-1 trial, is now standard (12, 13). Common adverse effects are hematologic (anemia, leukopenia, and thrombocytopenia), with grade 3–4 events in up to 13% and rare late complications (acute leukemia and myelodysplastic syndrome in ∼2%). Renal toxicity is minimal, aided by amino acid infusion for protection (12).

By integrating MRI-derived fat fraction and ^18^F-FLT PET proliferation metrics, clinicians may be able to monitor these early marrow suppression and renal changes during PRRT. These imaging biomarkers hold promise for predicting and mitigating long-term hematological and renal side effects, potentially guiding personalized treatment adjustments.

The aim of this study was, therefore, to assess whether the early renal and bone marrow effects previously identified in laboratory settings can be detected and studied, using MRI and ^18^F-FLT PET-MR in patients with neuroendocrine tumors receiving ^177^Lu-DOTATATE treatment.

## Materials and methods

### Study design

Ten subjects eligible for ^177^Lu-DOTATATE treatment were recruited to the study, expecting at least eight patients to complete five imaging visits over three treatment cycles. The study protocol was approved by both the ethics and radiation ethics committees (Regional Ethics Committee of Uppsala, Dnr 2010/177/3B and 2010/177/4B). The study was performed according to the principles of the Declaration of Helsinki, and all patients provided written informed consent. The trial registration number is EudraCT nr. 2009-012260-14.

The baseline imaging assessment was performed on the day of treatment 1 before the start of the treatment. Early treatment effects were evaluated on days 2 (48 h) and 7 (168 h) after infusion of 7.4 GBq (200 mCi) ^177^Lu-DOTATATE, referred to as treatment 1, and sustained treatment effects were assessed the day before treatment 2, which was performed 6–8 weeks after treatment 1 with the same therapeutic protocol. The final imaging was performed the day before treatment 4. Since most patients treated at the center were referred from other domestic regions and neighboring countries, no later imaging evaluations could be scheduled.

### Patients

Ten consecutive patients, four women and six men, were included in this study, scheduled to receive treatment with ^177^Lu-DOTATATE. Patients were enrolled between June 2018 and February 2019. Patients were considered eligible for PRRT treatment if they had NET expressing somatostatin receptors more than normal liver as established by somatostatin receptor scintigraphy, acceptable hematological status, and sufficient liver and renal functions. All patients had liver metastases, eight patients had lymph node metastases, and two patients had sporadic skeletal metastases. Eight patients were treated with somatostatin analogs, three with chemotherapy, three with interferon, and one with everolimus. No patients had previous PRRT treatment.

### Treatment protocol

On the day of treatment, 7.4 GBq (200 mCi) ^177^Lu-DOTATATE was administered as an infusion over 30 min. Four cycles within a 6- to 8-week interval were intended. However, for some patients, treatment had to be delayed as their laboratory status did not allow treatment at that time point.

To protect the kidneys from radiation exposure, patients received an extended infusion of basic amino acids (2 L Vamin 14 g/L) for eight hours, starting 30 min before ^177^Lu-DOTATATE treatment. As the amino acid infusion may cause nausea, patients were also administered antiemetics one hour before treatment (ondansetron 8 mg i.v. or palonosetron 250 μg i.v. in combination with betamethasone 8 mg i.v.).

A set of blood and urine markers was sampled on the day before treatment. Markers of particular relevance in this study comprised counts of white blood cells, granulocytes, platelets, and hemoglobin (Hb) and creatinine and cystatin C for eGFR assessments.

### Imaging

**Figure 1:**
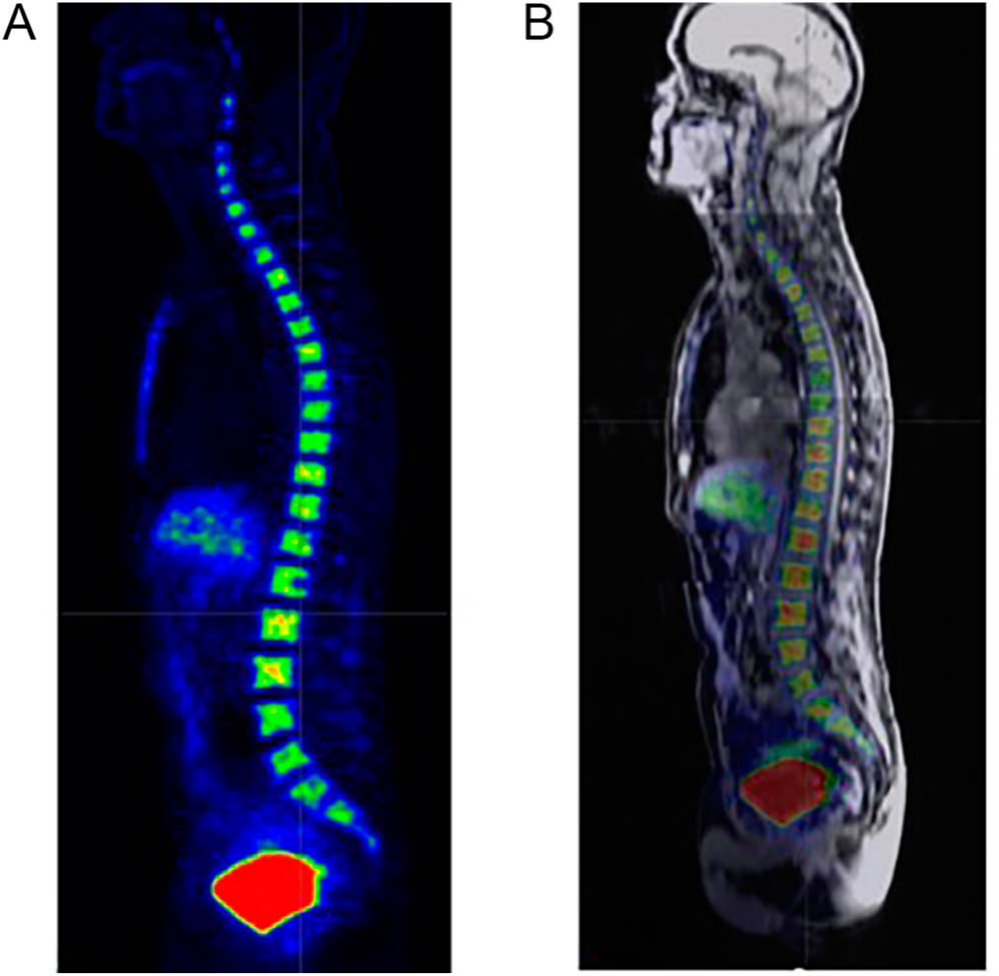
Sagittal image of pure ^18^F-FLT PET information (A) and fused sagittal ^18^F-FLT PET/MR image (B).

Imaging was performed using a combined PET/MR scanner (3.0T, Signa PET/MR, GE Healthcare) (Fig. 1). Subjects were instructed to ingest 250 mL of water before scanning to maintain kidney hydration. As part of the protocol, MRI-derived endpoints were assessed at all time points for all subjects, whereas ^18^F-FLT PET examinations were only conducted at baseline and day 2 after treatment 1 in a subset of patients (depending on tracer availability). The three imaging sessions scheduled on treatment days also included whole-body MRIs and contrast-enhanced abdominal MRIs for assessing treatment response (not covered in this report), replacing examinations otherwise performed using computed tomography (CT).

### Bone marrow

BM fat content as a marker for red-to-yellow BM conversion was assessed with MRI in vertebrae in the lower back region (typically T9 to S3). BM proton density fat fraction (BM-PDFF) maps were generated via a six-point Dixon technique, with base images acquired in free breathing (sagittal orientation, field of view (fov) 380 × 380 × 320 mm^3^, matrix size 224 × 224 × 32). A single central slice was selected for analysis, and individual vertebrae were manually delineated and evaluated in ImageJ (v. 1.51, U.S. National Institutes of Health, USA) (14).

For data presentation, the fraction of remaining red BM compared to the start of the treatment was estimated via 1 − ((PDFF_t_ − PDFF_t0_)/(0.85 − PDFF_t0_)), assuming a fat fraction of 85% in fully converted BM (14). A visual assessment of whether homogeneous or heterogeneous fat BM conversion was present within or between vertebrae was performed.

The BM uptake of ^18^F-FLT, a marker for tissue proliferation, was studied using PET. 4 MBq/kg ^18^F-FLT was administered i.v. one hour before the start of PET acquisition, and standardized uptake values (SUV_max_) were assessed (vertebrae C4 to S2, five bed positions, 3 min acquisition/bed, 2/28 iterations/subsets, reconstructed to fov 600 × 600 mm^2^, matrix size 192 × 192). PET image analysis was performed using Carimas (v2.9, Turku PET Centre, Finland). MRI and PET data are presented for the total volume of assessed BM and on individual vertebrae levels.

### Kidneys

**Figure 2:**
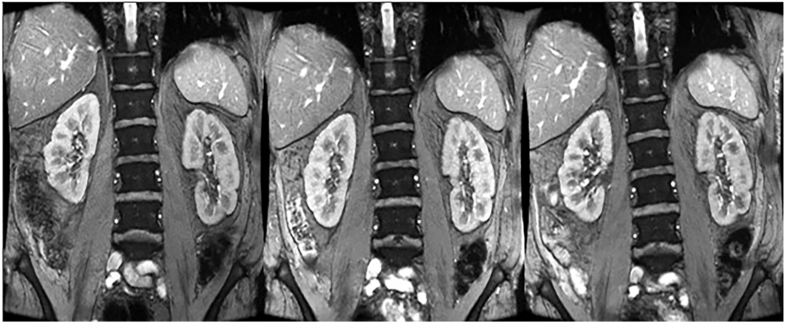
R2* images of the kidneys obtained from the same patients at three different time points: baseline, two days after PRRT treatment 1, and seven days after treatment PRRT treatment 2.

MRI markers were assessed as part of the renal imaging protocol. R2*-maps were generated via a multi-gradient echo sequence (coronal orientation, 14 echoes, DTE 2.0 ms, fov 320 × 320 mm^2^, slice thickness 5 mm, matrix size 224 × 224 mm^2^, five slices, one slice/breath-hold) (Fig. 2). Tissue R1 was determined from saturation recovery scans (SMART1MAP, coronal orientation, fov 440 × 440 mm^2^, slice thickness 5 mm, matrix size 224 × 160, single slice, breath-hold), whereas R2 was assessed from multi-spin echo images (coronal orientation, TE 30, 110, 179 ms, fov 320 × 320 mm^2^, slice thickness 5 mm, matrix size 224 × 224, five slices, five slices/breath-hold). Diffusion was analyzed from ADC maps (EPI, through-plane diffusion encoding, b-values 150, 300, 450 s/mm^2^, fov 320 × 320 mm^2^, slice thickness 5 mm, matrix size 112 × 112, five slices, five slices/breath-hold).

The renal parenchymal volume was calculated from T2-weighted high-resolution scans (axial orientation, free-breathing, respiratory-gated, 3D fast SE sequence, fov 340 × 340 × 150 mm^3^, matrix size 288 × 288 × 44). Finally, the renal arterial volume flow was assessed from phase contrast scans (breath-hold, pulse-oximetry triggered, 30 frames/cycle, fov 320 × 320 mm^2^, slice thickness 10 mm, matrix size 112 × 112, velocity encoding 100 cm/s) acquired perpendicular to each renal artery, approximately half-way between the kidney and the aorta.

For tissue markers, separate analyses were performed for the cortex and medulla, except for total renal parenchymal volume, which comprised both tissue types. All renal structures were manually delineated in ImageJ, whereas flow assessments in the renal artery were determined using Segment (v. 6.433, Medviso Imaging, Sweden) (15).

### Statistics

Hypothesis testing was performed via one-way ANOVA with repeated measures. Tests for change from baseline were conducted for early effects (on days 2 and 7) and for sustained effects (the day starting the second and fourth treatment cycles) with Dunnett’s multiple comparison correction.

## Results

Six of ten patients completed all four treatment cycles and the full imaging protocol. Three subjects withdrew during the first study week (two patients stated claustrophobia as their reason; the third patient completed scans at baseline and day 2 but withdrew consent after that). A fourth subject could not continue in the study following initial MR imaging since the status was considered too poor to allow treatment with ^177^Lu-DOTATATE.

### Bone-marrow assessments

**Figure 3:**
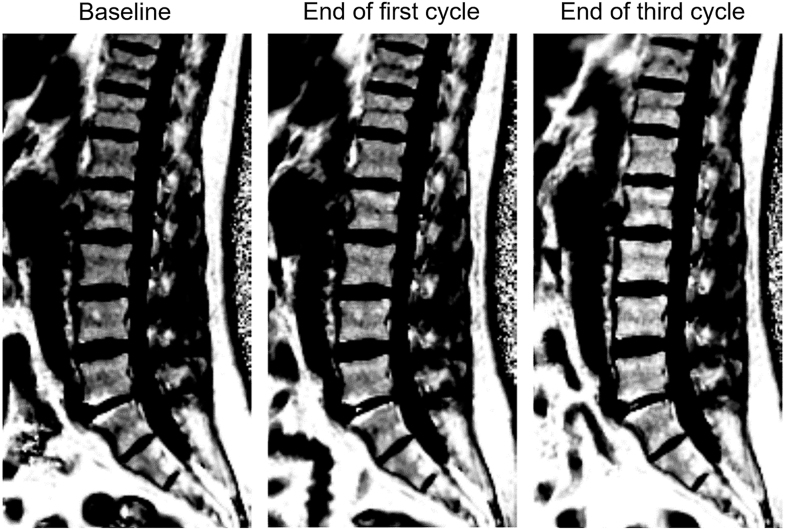
Sequential proton density fat fraction (PDFF) maps of the lower back region for one study patient. Images are acquired in the sagittal orientation using a six-point Dixon technique (voxel size 1.7 × 1.7 × 10 mm^3^). The increasing image intensity in bone marrow regions at the end of the first and third treatment cycles reflects bone marrow fat accumulation (BM-PDFF 53.0, 57.6, and 65.0%) and a hypothesized conversion of red bone marrow to yellow. Intensity increases are seen in all vertebrae. Other bright structures correspond to visceral and subcutaneous fat depots.

**Figure 4:**
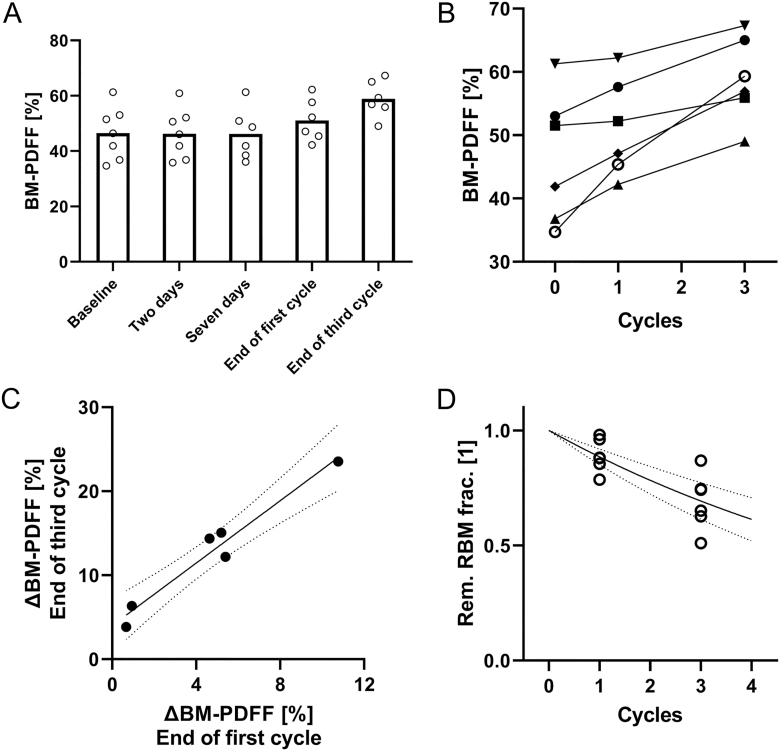
Bone marrow fat fraction (BM-PDFF) increases were found at the end of the first cycle (+4.6% ± 1.4%, *P* = 0.042) and before treatment 4 (+12.4% ± 2.8%, *P* = 0.013) (A). Baseline BM-PDFF levels and the increased level varied substantially between the study subjects (B). The BM-PDFF increase before treatment 4 correlated strongly with the increase at the end of treatment 1 (Pearson *r* = 0.98, *P* = 0.001) (C). By assuming a fat fraction of 85% for fully transformed tissue, the remaining red BM fraction can be approximated. Before treatment 4, on average, only 70% of the red BM remained in the observed region. Extrapolating the results to four cycles via a monoexponential function indicated that only 60% of the initial red BM mass may remain (D).

Baseline BM fat fractions (BM-PDFF) ranged from 34.7 to 61.3% in the study cohort. An increased BM-PDFF was found both after the first and third treatment cycles (+4.6% ± 1.4%, *P* = 0.042, and +12.4% ± 2.8%, *P* = 0.013, respectively) (Figs 3 and 4A). All individuals demonstrated an increased BM-PDFF during the study interval, ranging from +3.8% to +23.5% (Fig. 4B). A strong correlation was present between the increase after the first cycle and the increase after the third cycle (Pearson *r* = 0.98, *P* = 0.001, Fig. 4C). No early changes in BM-PDFF were found in the first week following treatment (+0.2% ± 0.4% at day 2; +0.4% ± 1.0% at day 7).

**Figure 5:**
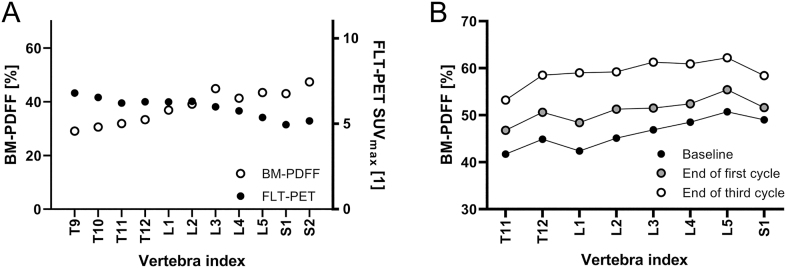
BM-PDFF and FLT SUV_max_ assessed in an individual vertebra in one patient. The gradient found in BM-PDFF levels was mirrored in a reversed gradient in ^18^F-FLT uptake data, illustrating the complementary information the two parameters gave (A). The bone marrow fat fraction (BM-PDFF) increases at the end of the first and third treatment cycles had a uniform impact on the vertebrae. The displayed data are averaged over all patients; only vertebrae covered by all examinations are included (B).

When investigating individual vertebrae visually, a gradient in BM-PDFF baseline levels was found, with higher values at lower vertebrae (Fig. 5B). The BM-PDFF increases following the first and third cycles were largely homogenous in each vertebra (Fig. 5B).

^18^F-FLT PET examinations were performed in two patients. On day 2, reduced tracer uptake was found in both subjects, −7.0% (SUV_max_ 4.8 to 4.4) and −3.9% (SUV_max_ 6.0 to 5.8). Compared to the BM-PDFF distribution over the vertebrae, similar but reversed gradients were found for tracer uptake data (Fig. 5A).

**Figure 6:**
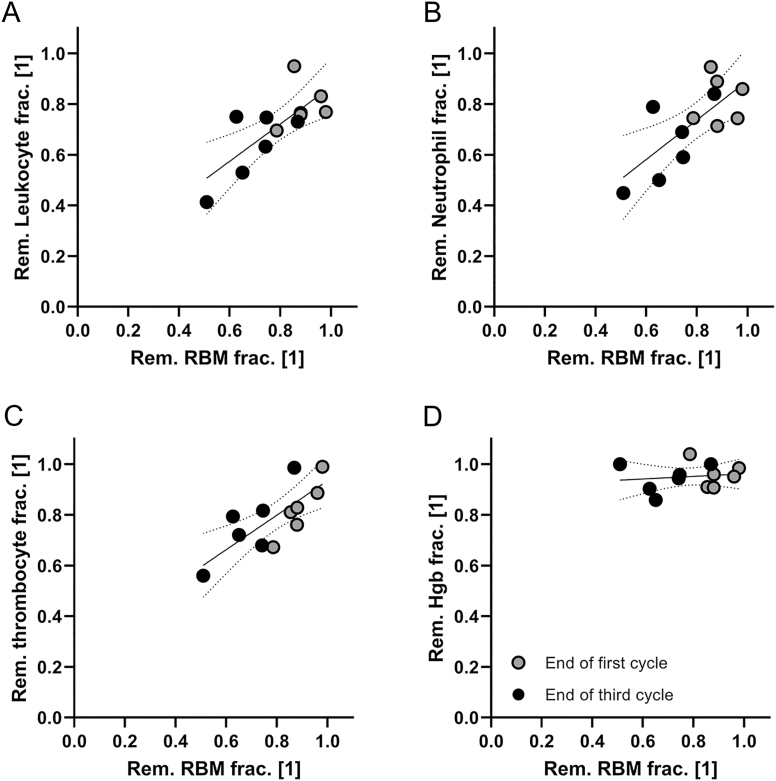
Relative reductions in white blood cells (A), granulocytes (B), platelets (C), and hemoglobin (Hb) (D).

Hematological analyses showed reduced white blood cell, granulocyte, and platelet levels after the first and third treatment cycles, whereas Hb levels were stable (Table 1). When the remaining fractions of hematological data were compared with the estimated remaining fraction of red BM, strong correlations for white blood cells (*r* = 0.75, *P* = 0.005, Fig. 6A), granulocytes (*r* = 0.72, *P* = 0.008, Fig. 6B), and platelets (*r* = 0.77, *P* = 0.004, Fig. 6C) were revealed.

**Table 1:** Laboratory counts of white blood cells, granulocytes, platelets, and hemoglobin and eGFR at baseline and after the first and third treatment cycles.

	Baseline	After first cycle	After third cycle
White blood cells (10^9^)/L	7.88 ± 1.33	6.08 ± 0.82	4.73 ± 0.54
Granulocytes (10^9^)/L	4.73 ± 1.10	3.75 ± 0.78	2.85 ± 0.50
Platelets (10^9^)/L	257.7 ± 29.4	209.2 ± 19.3	190.7 ± 16.9
Hb (g/L)	135.3 ± 3.5	129.5 ± 2.0	127.5 ± 2.7
eGFR (mL/min/1.73^2^)	79.8 ± 8.8	79.8 ± 8.6	79.8 ± 9.1

### Renal assessments

**Figure 7:**
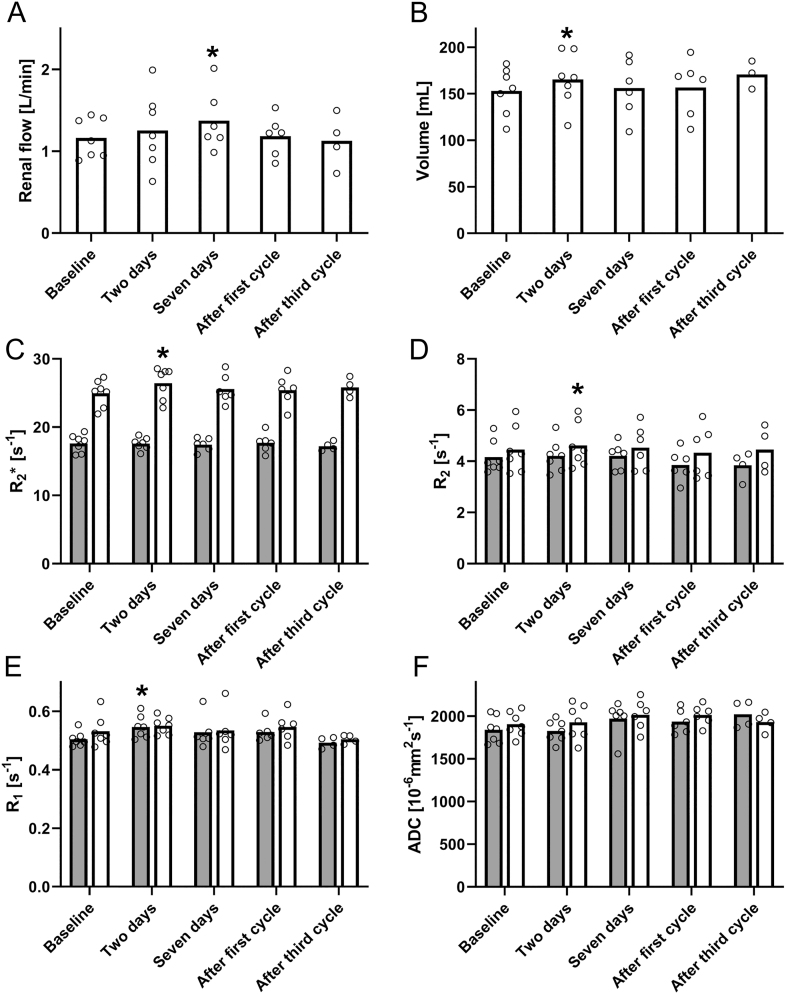
Changes in the renal arterial flow (A), parenchymal volume (B), cortical (dark) and medullary (light) R2* (C), R2 (D), R1 (E), and apparent diffusion coefficient (ADC) (F) at different time points. Data are presented as the average of the left and right kidney assessments, except for renal arterial flow, which is expressed as the sum of the left and right renal arterial flows. The asterisk (*) indicates significant difference.

Several changes in renal endpoints in the first week after treatment were identified. On day 7, the renal arterial flow was elevated (+0.26 ± 0.08 L/min, *P* = 0.038, Fig. 7A) compared to baseline. An increased renal parenchymal volume (+12.2 ± 3.5 mL, *P* = 0.024, Fig. 7B) was found two days after the first treatment. At the same time point, the medullary R2* and R2 were increased by 1.55 ± 0.36 s^−1^ (*P* = 0.013. Fig. 7C) and 0.21 ± 0.07 s^−1^ (*P* = 0.049, Fig. 7D), respectively, and the cortical R1 was increased by 0.032 ± 0.006 s^−1^ (*P* = 0.005, Fig. 7E). No sustained changes compared to baseline were found in assessments performed at the end of the first and third treatment cycles for any renal endpoint. At the corresponding time points, eGFR was also unchanged (Table 1).

## Discussion

In this study, noninvasive imaging techniques were used to investigate BM and renal adverse effects during and after ^177^Lu-DOTATATE treatment. Foremost, the study revealed a substantial increase in BM fat content. The magnitude of the increase exhibited a significant variation in the study cohort, and the implied level of reduction in hematopoietic bone marrow cells correlated strongly with the decrease in white blood cells, granulocytes, and platelets in the peripheral blood.

The study was not able to assess BM fat content after the last treatment cycle, but by extrapolating the presented data, it is indicated that the average reduction in red BM content at that time point could be as high as 40%, with some individuals having reductions of up to 60%. Whether the demonstrated tissue conversion is permanent or whether repopulation of red BM may occur following the end of therapy remains to be established. Since most patients treated at the center were referred from other regions within the country and neighboring countries, no follow-up imaging evaluations could be arranged.

The magnitude of the found conversion rates in our study is much higher than reports of BM fat fraction changes following external radiation treatment. In one study (14), a BM-PDFF increase of 0.4%/Gy was found following treatment of gynecologic, anorectal, and genitourinary malignancies. The mean of 12.8% BM-PDFF increase found in the present study was reached after three treatments, with the maximum dose to BM being 0.5 Gy per treatment, indicating a corresponding rate of over 8%/Gy. The type of radiation, systemic properties, and prolonged exposure are some factors that separate PRRT from external radiation therapy. It is also possible that relying on dose models assuming homogeneous BM tissue in the vertebrae may partly explain the fact that the percentage of fat conversion is higher in our material. As the red blood cells are mainly distributed along the sinusoidal vasculature (16), the dose may be underestimated for a short-range radiation source that largely stays intravascularly.

The lack of regional inhomogeneous pattern in BM-PDFF increase in the present data further implies that the radiation damage is of vascular origin and not due to the retention of the radio-labeled peptide in specific hotspots. However, studies covering larger regions of BM, also outside of the vertebrae, would be needed for confirmation.

Fat transformations of BM have also been reported in interventions other than ionizing radiation (16, 17, 18). In particular, restrictions in calorie intake can lead to an increase in BM fat content (17, 18). As no control group not undergoing PRRT was included in the study, it is possible that other factors than radiation might play a part. Other potential confounders could be changes in medication coinciding with the study start or lingering effects of previous therapies. Studying BM-PDFF levels before PRRT therapy may be a way to control this.

^18^F-FLT PET showed reductions in tracer uptake on day 2, corresponding to a similar magnitude as the increases in BM-PDFF after one treatment cycle in the two subjects investigated. When comparing ^18^F-FLT PET and BM-PDFF on a vertebral level, the complementary nature of the two techniques was demonstrated.

Among the renal assessments, changes from baseline were detected during the first week of therapy but not at later time points. This study’s most intriguing renal finding was an increase in R2* in the renal medulla detected on day 2, indicating that the region experiences hypoxia, as reflected by an increased fraction of deoxyhemoglobin (19). The incapacity to keep up with the metabolic demand, usually associated with increased levels of sodium reabsorption, is a central factor in renal medullary hypoxia (20). Identifying the underlying mechanisms that would lead to an increased metabolic demand and their possible link to radiation damage is beyond the scope of this work. However, the basic amino acid infusion should be mentioned as amino acids may also increase the metabolic stress on kidneys and induce mild tissue damage by themselves (21).

Renal medullary hypoxia is strongly associated with both acute and chronic kidney disease (20), but while our study indicates the presence of hypoxia, it is unclear if the magnitude or duration of the presumed hypoxia is sufficient to cause long-term harm. More extensive studies correlating early changes in tissue oxygenation to renal outcome data assessed at later time points than in the present study are required.

The increase in renal artery blood flow on day 7 is also consistent with an increased metabolic demand. The temporal mismatch between the two findings is not easily explained, but it should be mentioned that the R2* data on day 7 are disparate, with half of the study subjects demonstrating an even higher increase than on day 2 and half of the subjects demonstrating sharp decreases.

Renal parenchymal volume has been linked to renal function (22), where a larger parenchymal volume correlates to a better renal function and is hypothesized to be a marker for renal function decline at the pretreatment time points. No changes were detected at those time points in the present study. Early transient increases found on day 2 in the present study are much less explored. This could reflect induced alterations in reabsorption, urine production, or the presence of tissue edema.

Compared to the herein used method, the widely used DTPA (diethylenetriaminepentaacetic acid) scan primarily measures glomerular filtration rate (GFR) and renal clearance, offering a global functional assessment but limited anatomical details. DTPA is advantageous for simplicity, standardization, and quantitative GFR estimation, but it lacks sensitivity for detecting subtle parenchymal changes, microvascular alterations, or early fibrosis.

The main drawback of this study was the small number of patients who followed the entire study protocol. However, the findings of increased bone marrow fat are so significant across all patients that it is considered reliable. Regarding the results from the two patients who underwent ^18^F-FLT PET-MR, no definitive conclusions can be drawn; instead, they merely support the primary MRI finding. Another limitation of our study was the inability to perform MRI, clinical, or laboratory follow-up investigations after the final treatment. This was primarily due to logistical constraints, which prevented us from determining whether the observed changes in bone marrow and blood counts were permanent or transient. All but one of these patients were international – seven from Norway and two from Ireland – and had traveled to Sweden exclusively for PRRT treatment. Consequently, we did not obtain ethical approval to conduct MRI, clinical, or laboratory follow-up investigations.

## Conclusions

The hematological effects observed with laboratory tests after ^177^Lu-DOTATATE treatment in neuroendocrine tumor patients can be detected by MRI as an increase in bone marrow fat content. This finding was supported by decreased FLT uptake on ^18^F-FLT PET-MR, indicating decreased bone marrow cell proliferation. MRI can also identify renal effects associated with this therapy.

## Declaration of interest

Angeliki Dimopoulou Creusen has no conflict of interest. Håkan Ahlström is a co-founder and employee of Antaros Medical AB. Edvin Johansson and Per Hagmar are employees of Antaros Medical AB. Dan Granberg has no conflict of interest.

## Funding

This study was funded by A1M Pharma (now Guard Therapeutics), Antaros Medical AB, grants from the Swedish state under the agreement between the Swedish government and the county councils, the ALF agreement, and the Swedish Cancer Society (Håkan Ahlström 23 3123 Pj 01 H).

## Author contribution statement

EJ, HA, DG, and PH conceived the study. DG included the patients. ADC was responsible for writing the manuscript. All authors analyzed the data and revised the manuscript. ADC and EJ made equal contributions and share the first authorship, and HA and DB made equal contributions and share the senior authorship.

## Ethics

The study protocol was approved by both the ethics and radiation ethics committees (Regional Ethics Committee of Uppsala, Dnr 2010/177/3B and 2010/177/4B). The study was performed according to the principles of the Declaration of Helsinki, and all patients provided written informed consent. The trial registration number is EudraCT nr. 2009-012260-14.
